# Enhanced Field-Based Detection of Potato Blight in Complex Backgrounds Using Deep Learning

**DOI:** 10.34133/2021/9835724

**Published:** 2021-05-16

**Authors:** Joe Johnson, Geetanjali Sharma, Srikant Srinivasan, Shyam Kumar Masakapalli, Sanjeev Sharma, Jagdev Sharma, Vijay Kumar Dua

**Affiliations:** ^1^School of Computing & Electrical Engineering, Indian Institute of Technology Mandi, Kamand, H.P., India; ^2^BioX Center, School of Basic Sciences, Indian Institute of Technology Mandi, Kamand, H.P., India; ^3^ICAR-Central Potato Research Institute, H.P., Shimla, India

## Abstract

Rapid and automated identification of blight disease in potato will help farmers to apply timely remedies to protect their produce. Manual detection of blight disease can be cumbersome and may require trained experts. To overcome these issues, we present an automated system using the Mask Region-based convolutional neural network (Mask R-CNN) architecture, with residual network as the backbone network for detecting blight disease patches on potato leaves in field conditions. The approach uses transfer learning, which can generate good results even with small datasets. The model was trained on a dataset of 1423 images of potato leaves obtained from fields in different geographical locations and at different times of the day. The images were manually annotated to create over 6200 labeled patches covering diseased and healthy portions of the leaf. The Mask R-CNN model was able to correctly differentiate between the diseased patch on the potato leaf and the similar-looking background soil patches, which can confound the outcome of binary classification. To improve the detection performance, the original RGB dataset was then converted to HSL, HSV, LAB, XYZ, and YCrCb color spaces. A separate model was created for each color space and tested on 417 field-based test images. This yielded 81.4% mean average precision on the LAB model and 56.9% mean average recall on the HSL model, slightly outperforming the original RGB color space model. Manual analysis of the detection performance indicates an overall precision of 98% on leaf images in a field environment containing complex backgrounds.

## 1. Introduction

Early and late blight diseases are a common occurrence across regions where potato (Solanum tuberosum L.) is cultivated. Blight is a common foliage disease of potato that starts as uneven light green lesions near the tip and the margins of the leaf and then spreads into large brown to purplish-black necrotic patches as reported by Arora et al. [1]. Blight causes premature defoliation and eventually incites tuber rot of potato. As noted by Haverkort et al. [2], unchecked blight could destroy the entire crop within a week under conducive conditions. Thus, blight in potato could bring disastrous consequences, particularly to farmers with marginal landholding who grow potato as cash crops [3].

In most developing countries, detection and identification of blight are performed manually by trained personnel scouting the field and inspecting potato foliage. This process is tedious and in some cases impractical, due to the unavailability of a disease expert in remote regions [4]. On the other hand, the recent advances in image processing for rapid and automated disease identification using images of plant leaves [5–7] can make the process far more efficient and timely. In the recent past, a system has been proposed to identify the severity of potato late blight disease from field images using fuzzy *C*-means clustering [8] but with few images. Using 300 images as a training set, another work [3] has attempted potato disease detection using segmentation and multiclass support vector machine. These datasets do not incorporate time-varying illumination and are usually taken at a fixed time corresponding to the best illumination. Usually, methods developed using small datasets do not perform well in field environments due to the large variations in illumination, focus, resolution, underlying feature size, and presence of occluding objects in the images.

More recently, the task of classification and detection in images has been dominated by various flavors of neural networks (NNs), especially with the advent of deep NNs [9–14]. It has been well-accepted that deep learning models perform quite well in image classification and detection compared to traditional image-processing algorithms [15]. The process of trial and error for fine-tuning traditional image processing models to obtain the representational features of objects becomes rapidly complicated as the number of classes increases. On the other hand, a neural network learns complicated underlying patterns specific to a certain class of object without any manual intervention. A classification model using convolutional neural network (CNN) for distinguishing 58 classes of healthy and diseased plant dataset was developed by Ferentinos and Konstantinos [16]. Arsenovic et al. [17] have improved the plant disease classification by increasing the training dataset, which has images of leaves in field conditions. Deep NNs have the potential to quickly detect an object from a complex image, which makes them suitable for smart phone applications [7]. However, the training process in deep NNs is computationally expensive where the network parameters are iteratively fine-tuned to improve the mapping between a set of training input images and the desired outputs [9]. Therefore, such methods have become popular only with the concomitant advances in graphics processing hardware.

In the context of an image comprising a potato leaf amidst a complex background, the classification process has a binary outcome; i.e., it determines if the overall image reflects disease or not. Detection, on the other hand, goes one step further and demarcates the specific patch or patches on the leaf that contain the signature of blight. Region-based deep CNN (R-CNN) [18] is an object detection method that is trained to propose regions by exhaustively searching the image after it has been transformed through several convolution layers. For the purpose of object detection, architectures like YOLO [19], SSD [20], Faster R-CNN [21], and Mask R-CNN [22] are recent methods, with Mask R-CNNs giving a better overall performance. For the R-CNN architectures, the residual network with 50 layers (ResNet-50) is usually used as a backbone. Other applications of CNN in agriculture include Zhang et al. [23] who have used global pooling dilated CNN for better segmentation and classification of cucumber leaf disease, while CNN-based regression has been used to estimate soybean leaf defoliation with the aid of real and synthetic images [24].

The various transformations that an image undergoes as it traverses a deep CNN can sometimes be understood by visualizing the output of individual convolutional layers. The output is termed as the feature map or activation map and can be visually correlated to the input image. Each convolutional layer is a set of functions that applies some transformation to the image, behaving as a filter. The feature map aids in relating the learned filter with the performance of the model and using the learned filter to improve the performance as discussed in [25]. Lee et al. [26] have reported such studies in plants where the different orders of venation provide better representative features than the outline shape of a leaf when considering the hierarchical transformation of features from lower-level to higher-level abstraction for species classes.

In addition to the choice of appropriate NN architectures, preprocessing the image data can contribute towards obtaining better detection or classification. For example, it has been observed that a color spectrum provides better results than grayscale for object detection by deep learning models [7]. A color space or color model is a mathematical transformation to project a set of primary colors to a different range of colors [27]. An investigation of the influence of different color spaces to improve the deep learning model performance has been conducted for the traffic light detection system [28]. A comparative study for different color spaces using deep learning-based automatic segmentation system has been discussed in [29]. Disease region segmentation of paddy crop using Mask-RCNN on different color space images is analyzed in [30]. Robustness and accuracy of the segmentation of foliar disease spot images using region growth and comprehensive color features have been explored in [31].

The objective of this work is to develop a Mask R-CNN-based model to detect the blight symptoms on an infected potato leaf, which can eventually be deployed on a cell phone. Mask R-CNN [22] is chosen because it utilizes a feature pyramid network (FPN), allowing it to grasp semantically relevant features at different resolution scales. The region proposal network (RPN) scans the entire top-bottom pathway of the FPN for feature maps containing required objects and proposes regions of interest (ROI). This enables prediction of relevant classes, bounding boxes, and mask for the region or patch. These methods of Mask R-CNN force different layers in neural network to learn features across multiple scales, making it robust to several environmental variations in the image. The model learns features from visual characteristics such as the shape, color, texture, and venation of a potato leaf and blight disease for different training data.

**Figure 1:**
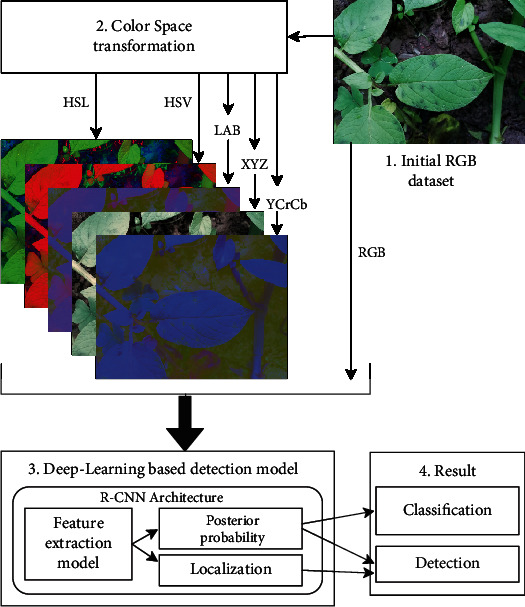
Overview of the deep learning-based potato disease classification and detection method in this work.

The emphasis on detection rather than classification is because simple classification into healthy or unhealthy categories can be misleading due misclassification of soil patches in the background as disease. To improve blight detection, we also investigate preprocessing the data to include different color space images.  Figure 1 conveys the overview of the method proposed in this work. We have converted the RGB color space dataset to five other color spaces, namely, HSL, HSV, LAB, XYZ, and YCrCb and created a separate Mask-RCNN model for each color space. The model uses transfer learning or stored knowledge of a pretrained Mask R-CNN model on the Microsoft Common Objects in Context (MS COCO) dataset [32] as the initial condition for the training process. The performance of the networks across the different color spaces is compared in their ability to automatically detect infected potato leaves and disease patches in complex field images.

## 2. Materials and Methods

### 2.1. Data Acquisition

The choice of data used for training a CNN has a very strong impact of the effectiveness of the model in different situations. Factors such as the characteristics of the imaging sensor, the imaging protocol followed, illumination variation due to time of the day, shadows due to nearby objects, occlusion, and complex background information all need to be carefully considered to create a model that can be successfully applied to field-based imaging. In order to maximize the diversity of training data, a set of 1840 field-based images of potato leaves was acquired for this work across different states in India by field personnel deputed under the FarmerZone project [33].

The dataset comprises images of healthy potato leaves as well as leaves affected by both early and late blights. As one of the objectives was to develop a model that would be accessible to a larger group of small-scale farmers, it was determined that the choice of imaging sensors should include low-end cellular phones. Therefore, the potato leaf dataset contains images of resolutions of 3072 × 4096 pixels (552 images), 3120 × 4160 pixels (922 images), and 2448 × 3264 pixels (366 images) due to inherent differences in the sensors of the different smart phones used for data collection.

**Figure 2:**
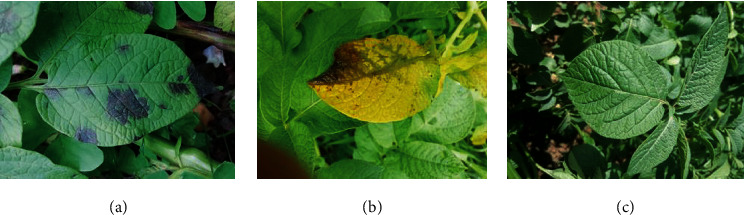
The complex background dataset used in this study: (a) multiple disease patches on a single leaf; (b) single disease patch on a discolored leaf; (c) healthy leaves.

The images are heterogeneous, having been taken from different locations within the field at different times of the day, typically between 11 am and 2 pm. Each image can contain several leaves, soil, and weeds in the background apart from the primary infected/healthy potato leaf. This variation aids the generalization of the deep learning model. All images were captured in natural light with the camera flash always turned off and without any additional optical or digital zoom. Sample images of healthy leaves and leaves affected with blight are shown in Figure 2.

### 2.2. Data Curation

The potato leaf images obtained using smart phones are in the RGB format, which is similar to the human perception of the light spectrum as a combination of the primary colors—red, green, and blue [34]. While there is potential for improved image segmentation using other color spaces, there is no general opinion on the best choice of color space for image segmentation. Therefore, all the RGB images were converted to five color spaces (HSV, HSL, XYZ, LAB, and YCrCb) using Open Computer Vision Library [35], creating additional 5 datasets. In the RGB dataset, one or more blight spots on each potato leaf in the foreground are manually demarcated into patches for creating the ground-truth dataset. The process of demarcating or segmenting the images was carried out by three personnel, two nonexperts under the guidance of an agricultural expert. The ground-truth values and labels are kept the same for all the images in different color space datasets. To reduce the annotator's bias and variance [36] during ground-truth annotation, the following steps were taken:

- The expert first demonstrated the protocol for segmentation of patches on foreground, the edges to be considered, and how tightly the polygon should be drawn
- For 50 randomly selected images, the expert and the nonexperts all annotated according to the prescribed procedure. The value of Cohen's kappa [37] found across the three annotators was 0.92, and level of agreement was found to be very good. Thereafter, 5825 blight patches, 1779 infected leaf patches, and 211 healthy leaf patches were created from the 1840 input images.  Table 1 provides the details of the total dataset and its annotation count. To create and validate the disease detection model, the dataset of each color space was further split into 2 sets containing approximately 80% and 20% data, respectively

### 2.3. Mask R-CNN-Based Detection Model

**Figure 3:**
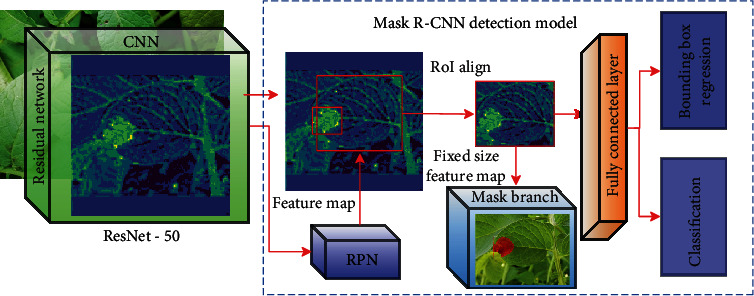
Block diagram of a model architecture for the implemented Mask R-CNN models.

The detailed block diagram of Mask R-CNN used in this work is shown in Figure 3. Mask R-CNN is an extension of Faster R-CNN [38], with an additional forking to a prediction segmentation mask on each RoI, in parallel with the already available branch for classification and bounding box regression. In this work, further tuning of the original Mask RCNN includes the use of ResNet-50 as backbone architecture with RPN anchor scales set to 32, 64, 128, 256, and 512 and the anchor aspect ratios set to 1 : 2, 1 : 1, and 2 : 1. This follows from manual observation of the training dataset, which shows that the various demarcated patches vary in this selected range of pixel values and aspect ratios.

Regarding the choice of ResNet-50 as the backbone, it may be noted that deep CNN is prone to problems like vanishing gradients and the curse of dimensionality [14], with an increase in the number of layers. To avoid this degradation problem for a deeper network, skip connections (identity connections) or residual connections are used. The residual connection is a “shortcut” module, whereby the weight/convolutional layers are skipped and the input is added through an identity function before the final ReLU activation function. It is observed that during backpropagation, larger gradients are available for initial layers leading to faster learning because of skip or residual connection. ResNet-50 has 50 layers arranged in five stages with a total of sixteen residual blocks. In each residual block, the convolutional layer is followed by a batch normalization layer and a ReLU activation function. The ResNet-50 model generates 256, 512, 1024, and 2048 feature maps from the second, third, fourth, and fifth stages, respectively.

Each color space dataset is used for training a separate Mask R-CNN detection model. In a preprocessing step, the input images are downsampled to 1024 × 1024 pixels. For each color space model, the mean value of each channel of the respective color space, calculated separately from the training dataset, is set in the configuration file of the program [38]. Pretrained weights of the MS COCO dataset have been used for the initial training of the model as attempts to train from scratch did not yield significant detection even after 70^th^ epoch for all of the color space datasets, probably due to the small dataset. On the other hand, the application of transfer learning towards classification of potato leaf disease was shown in [39, 40]. To optimize the network weights, the stochastic gradient descent optimizer with momentum fixed at 0.9 was used. A fixed learning rate of 1*e*-4 was set for optimum learning. The maximum number of epochs was set to 100, and iterations per epoch were set to 712 corresponding to a batch size of two images per iteration.

### 2.4. Computing Resources Utilized

The training and testing of the model were performed on a CentOS 7 Linux workstation equipped with one Intel Xeon Processor CPU (96 GB RAM), accelerated by one Nvidia GeForce GTX 1080 Ti GPU (11GB Memory). The model is implemented in the Keras 2.2.4 deep learning open-source framework with the TensorFlow-GPU 1.8.0 backend using Python 3.6. The detection model on each color space took an average of 25 hours for training.

For creating the ground-truth dataset VGG Image Annotator (VIA) [41], a standalone software was used for the manual annotation of the blight and leaf patches in the image. It allows a rectangular- and polygonal-shaped area to be annotated, which is useful for training Mask R-CNN.

### 2.5. Model Evaluation Metrics

In computer vision, standard metrics like precision and recall are used for performance evaluation of binary classification [42]. This is obtained from a confusion matrix that summarizes the performance of a classifier for a given test dataset. The four components of the 2 × 2 confusion matrix for any binary classifier are true positive (TP), true negative (TN), false positive (FP), and false negative (FN). The correct classification of an image containing disease would count as a TP, while an incorrect classification as a healthy image would count as a FP. The performance of the classifier is then obtained by

(1) $$\begin{array}{c} \text{Precision}=\frac{\text{TP}}{\text{TP}+\text{FP}}\;\text{Recall}=\frac{\text{TP}}{\text{TP}+\text{FN}}. \end{array}$$

For the performance assessment of the object detection model, both the correct classification and the precise location of the disease patch in the image should be taken into account. To do so, concepts such as intersection over union (IoU) and the average precision (AP) were introduced in the Pascal VOC challenge [43]. The IoU metric determines the correctness of the patch detection by taking into account how closely the predicted instance (PI) fits the ground-truth instance (*G*). IoU is the measure of overlap between *G* and PI boundaries given by

(2) $$\begin{array}{c} \text{IoU}\left(G,\text{PI}\right)=\frac{G\cap \text{PI}}{G\cup \text{PI}}. \end{array}$$

**Figure 4:**
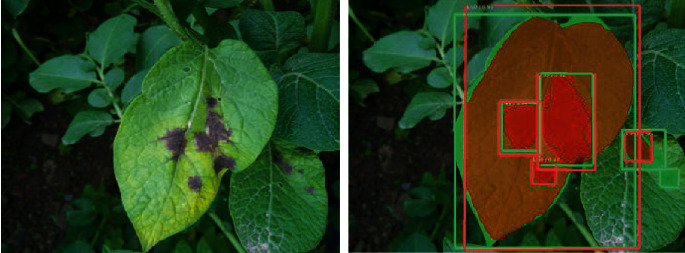
An example to describe IoU on the object with and without annotation.

The IoU threshold is taken to be 0.5 as a common practice, whereby if the IoU value of detection is greater than 0.5, then the PI is considered as a TP, or else it is taken as a FP. This is illustrated using a sample test image shown in Figure 4, where green color masks and bounding boxes represent the human-annotated ground truth while red color masks and bounding boxes represent the predictions by the detection model. For the sample image shown in Figure 4, the confidence score and IoU for the infected leaf are 100% and 93%, respectively.

In addition to the boundaries of the PI, the algorithm also provides a confidence level for the PI. The AP is a metric that incorporates the confidence level of prediction and IoU into the calculation of precision using the area under precision-recall curve. Mean average precision (mAP) is mean of AP across the different categories or classes, which are detected, and summarizes the performance of a detection model.

## 3. Results and Analyses

### 3.1. Disease Detection

**Figure 5:**
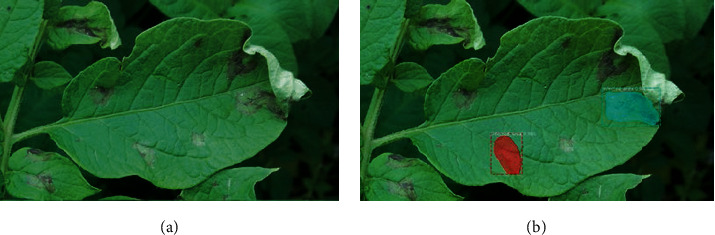
(a) Sample test RGB image and (b) output image for the trained two-class Mask R-CNN model.

**Figure 6:**
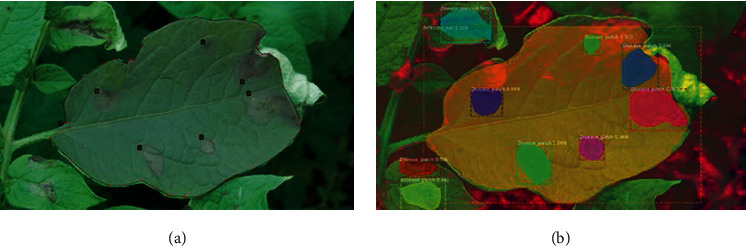
Sample RGB image: (a) human annotated foreground regions; (b) patches inferred by the 4-class HSL model.

**Figure 7:**
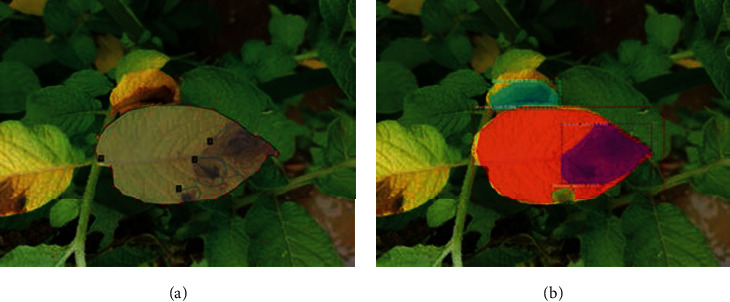
Sample RGB image: (a) with annotated foreground regions; (b) patches inferred by the 4-class RGB model.

The performance of the disease detection model, when tested on the ground-truth potato leaf dataset, is calculated according to the metrics defined in Section 2.5. A separate model is created for each color space. Even within each color space, there are two types of Mask R-CNN models:

- Two-class model: this involves the detection of only potato blight patches, while the rest of the image is considered as background. This kind of demarcation is a natural first step where it is expected to detect only blight disease patches from the input image. However, once the model was trained over the entire dataset, it was found that several blight patches were not detected and that a few soil patches were misclassified as blight. A sample test image from the RGB dataset (Figure 5(a)) contains nine disease patches spread across three different leaves. Figure 5(b) shows that the two-class model has detected only two disease patches out of nine clearly distinguishable disease patches
- Four-class model: as a means to improve the performance of the detection model, a second experiment was performed in which the Mask R-CNN model was trained to detect 4 classes: blight disease patches, infected leaves, and healthy leaves, in addition to the background (Figures 6 and 7).

For both models, the ground-truth criteria were kept uniform for all the images. The aim of this second model was to increase blight disease patch detection and reduce the FP due to misclassification of soil as disease, by the inclusion of a postprocessing step that checks for the intersection of the disease patch with the leaf patch. Nevertheless, it was seen that the performance of the four-class model was superior to that of the two-class model even without any additional postprocessing. The performance scores for the 2-class and the 4-class detection models are compared in Table 2 with respect to different color spaces.

Among the two-class Mask R-CNN models for different color spaces, LAB color space has the best mAP (80.1%) and mAR (55.6%) values. The 4-class detection model shows a slightly improved mAP (LAB) and mAR (HSL) performance metrics of 81.4% and 56.9%, respectively. It was observed that HSL, LAB, and YCrCb color space models could perform better than RGB color space model overall and specifically for disease patch detection. Inference times are the least for the detection model trained on HSL and HSV color spaces. These results are in line with the latest leaderboard on the COCO website [44], which publishes the performance results for different models on the COCO dataset with 91 categories. The highest mAP (IoU = 0.5) is 60.6% for a model trained on the dataset of broccoli category (closest to potato leaves).

Further investigation into the performance of different four-class detection models by manually comparing the test image data to the model outputs shows that the performance of the disease detection model appears far better than the mAP and mAR values reported in Table 2. This surprising outcome can be understood if we delve into the ground-truth labeling procedures. The images taken from the field have many complex regions due to fuzziness of image, partially occluded disease patches, and disease patches on the stem. Many disease patches that fall into these categories were not annotated while creating the ground-truth dataset. Also, for the human annotator, there is often no clear distinction between the foreground and background features, whether for disease patches or the leaves. The human annotator, for example, has annotated (shaded region shown in Figures 6(a) and 7(a)) only clearly distinct features of disease or leaf patches. However, our trained models have correctly predicted several unlabeled disease patches in the background as disease (Figures 6(b) and 7(b)). Since these “vague” disease patches have not been labeled in the ground-truth dataset, they end up lowering the mAP and mAR parameters, despite the correct classification by the model. Hence, the ground-truth annotation might need to be more inclusive of disease patches to better represent the performance score.

### 3.2. Manual Analysis of the Detection Model

Considering the challenges of ground-truth labeling, we attempted a more realistic quantification of disease patch detections by manually verifying the outputs of the 4-class model (Table 3). The correct disease patch predictions were categorized into two true-positive categories: TP1 reflects the correct detections that match the ground truth while TP2 reflects correct disease detections that have not been annotated in the ground truth. The same exercise is carried out for the infected leaf and healthy leaf classes also. Table 3 summarizes the results of manually determining the detection performance on the test dataset, for all color spaces. It can be inferred that among the six color space models, the model trained on HSL color space has the best disease detection with 464 (TP1) patches detected. The LAB and YCrCb color space trained models have the best combined four-class performance metrics of 98.6% (combined precision) and 85.8% (combined recall), respectively. The YCrCb model shows maximum true disease detection (TP1 + TP2) of 647 disease patches. The infected leaf patches were detected better by the HSV color space model with 341 true detections. It was observed that HSL, HSV, LAB, and YCrCb models performed better than the RGB color space model for the detection of disease patch and infected leaf. In all color space instances of the 4-class model, very few FP are observed for disease patch class while most of the FP in the infected leaf class are misclassifications of a healthy leaf.

### 3.3. Analysis of the Role of Color Spaces

**Figure 8:**
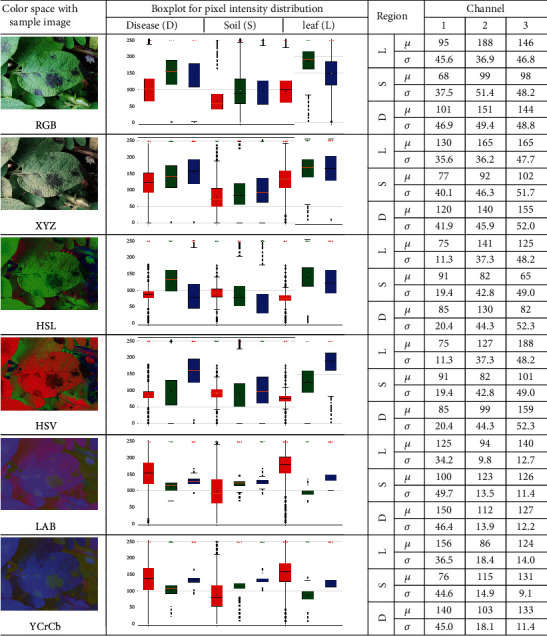
Sample image and box plot of the pixel intensities of the three channels for each color space, for the disease, soil, and leaf patches (channel 1, channel 2, and channel 3 are represented by red, green, and blue color, respectively in the box plots). Mean (*μ*) and standard deviation (*σ*) corresponding to the box plots are also provided for numerical comparison.

Hadji et al. [45] have previously shown that the histogram of image intensities is used broadly for recognition and retrieval in an image database. For a better understanding of the effect of each color space on the potato leaf dataset, the histogram trends of various color components in the image can be observed [46].  Figure 8 shows a histogram analysis on thirty randomly selected potato leaf images with blight symptoms. Each image was of 2448 × 3264 pixels and further divided into image patches of 200 × 200 pixels. All patches were manually labeled into classes of blight disease, healthy leaf, soil, and background. The count of patches for each class was as follows: the blight disease class contained 844 patches, the healthy leaf class contained 2216 patches, the soil class contained 102 patches, and patches that did not meet the predetermined patch size and features were discarded. The pixel intensity distribution for patches of disease (D), soil (S), and leaf (L) regions in the form of a box plot with the mean (*μ*) and the standard deviation (*σ*) for each channel of the color space is shown.

It is observed from Figure 8 that each of the channels of the RGB color space shows pixel distribution with a large spread that overlaps with the adjacent regions. This is due to varying illumination conditions across images, which equally affect the R, G, and B channels. Only for channel 2 is there some separation between the distribution of disease and leaf regions. This color information might be used by a deep learning model for classification. Similar to the RGB color space, the XYZ color space has a wide distribution of intensity values for all the components. Here, channel 2 has better separation between soil and leaf regions.

Conversion to HSL from RGB color space restricts the range of certain components such as hue, which are illumination independent. Therefore, the hue component is expected to have a narrow range for all regions. Thus, it can be observed that although each of the HSL channels' distribution overlaps across all regions, the overlap is minimum for the hue channel. The many outliers might still make classification difficult using only hue information. Similar to HSL, the HSV color space has the same hue information. Compared to HSL, the HSV has more spread in pixel intensity distribution for channels 2 and 3. This might lead to reduced performance of the detection model. In the case of LAB, channel 1 (lightness) varies according to lighting conditions. The component a∗ and b∗, represented by green and blue boxes, respectively, are the green–red component and a blue-yellow component of the image. From Figure 8, it can be observed that a∗ and b∗ components have a narrow range of pixel intensity values. Here, the a∗ component shows separation in values for leaf, soil, and disease regions. Similarly, the b∗ component has a very narrow overlapping area. This clear segregation in the a∗ and b∗ components could help in better classification and detection models. For the case of YCrCb, blue-difference chroma and red-difference chroma have narrow spreads for different regions. The red box represents the luma component; green and blue boxes represent blue and red-difference chroma, respectively. In channel 2, the soil and the leaf regions are distributed apart from each other while all other channels overlap in their distributions for the different regions. Thus, the histogram analysis helps to understand the color complexity of different patches/regions and it is observed that the color information will solely not lead to good segmentation between disease, soil, and leaf regions. Higher-level features like the texture of the disease region and leaf venation will need to be used by the deep learning model for segmentation, in addition to color.

### 3.4. Feature Map Observations

**Figure 9:**
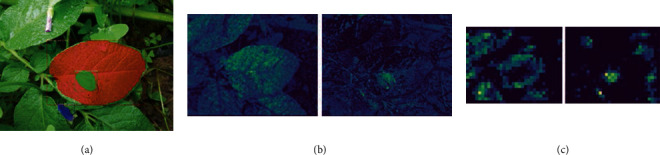
(a) Sample output image, corresponding visualization of the leaf, and disease-relevant feature map for (b) 2c and (c) 5c layers of the RGB color space detection model.

The characterization of blight disease, soil, and leaf regions by the CNN can be observed from the deconvolutional layers. The deconvolutional network maps the feature activity back to the input pixel space by using the same components of the convolutional layer (filtering, pooling) in the reverse order [25, 26]. The feature maps of the different stages of ResNet, trained for four-class detection include a number of relevant ones showing features of leaves and disease patches.  Figure 9(a) shows the sample output image for the RGB color space detection model. Figures 9(b) and 9(c) both show the visualization of the leaf feature and disease patch feature side-by-side for the 2c and the 5c layer, respectively. From Figure 9, it was inferred that the leaf features are well learned. The activation in the 5c layer shows that along with features of disease patch, features of soil patch have also been learned by the detection model. Overall, the 36^th^, 12^th^, 31^th^, and 28^th^ feature maps of the 2^nd^ to the 5^th^ layers were strongly related to disease, flower in the background, infected leaf, and healthy leaf, respectively. The learning of leaf venation could be properly observed in the feature maps.

**Figure 10:**
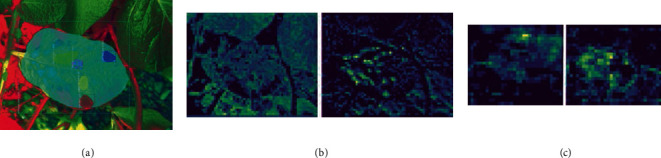
(a) Sample output image, corresponding visualization of the leaf, and disease-relevant feature map for (b) 4c and (c) 5c layers of the HSL color space detection model.

It is interesting to observe that for the model trained with HSL color space dataset, learning and extraction of the finer leaf and disease patch features were observed in the visualizations for the second to the fifth stage of the model. From the leaf feature maps, it could be inferred that a leaf's feature is better learned when it is in proper camera focus. The feature maps for the sample HSL output image are shown in Figure 10(a) with the fourth and the fifth stages shown in Figures 10(b) and 10(c). Here, the diseased patch is clearly learned apart from the background or the soil patches.

**Figure 11:**
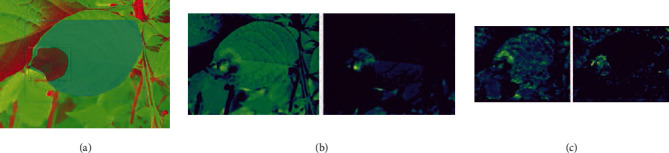
(a) Sample output image, corresponding visualization of the leaf, and disease-relevant feature map for (b) 2c and (c) 4c layers of the HSV color space detection model.

Similar to HSL, the HSV color space detection model has learned the leaf venation and disease patch structures clearly, which are visible in all the leaf feature maps of different stages for the model shown in Figures 11(b) and 11(c). Here, a total of 54 and 43 feature maps have a strong correlation with the diseased patch and leaf feature, respectively.

**Figure 12:**
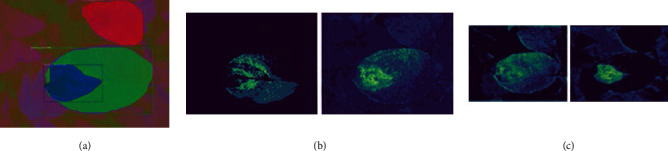
(a) Sample output image, corresponding visualization of the leaf, and disease-relevant feature map for (b) 2c and (c) 3c layers of the LAB color space detection model.

The sample LAB color space image shows that the disease patch (dark-blue color mask), the infected leaf (green color mask), and the healthy leaf (red color mask) have all been detected. From the feature maps shown in Figure 12(a), it is observed that the diseased patch, the infected leaf, and the healthy leaf features are learned separately. The YCrCb color space model detects the various classes similar to the LAB color space model with a sharp distinction between the three classes.

## 4. Discussion

The primary aim of this work is to deliver blight advisories to potato farmers in a timely and automated manner. As blight spreads fairly rapidly, farmers are advised to spray fungicide as soon as blight occurrence is detected. Therefore, in addition to successfully detecting true occurrences of blight, the blight model should also minimize the number of false-positive detections. Otherwise, it can lead to unnecessary spraying of fungicide and higher input costs to farmers. False positives can be a problem when using simple binary classification since the model might misinterpret the background soil patches as occurrences of blight and give a false alarm. Therefore, both a 2-class detection model and a 4-class detection model are explored in this work.

The use of various color space transformations for preprocessing the data enables higher detection accuracy by circumventing the variations in lighting conditions on the field. While this work has presented the performance of individual color models, one may easily create a consensus system using multiple color models in parallel, to further enhance the detection. This kind of software and algorithmic approach to processing RGB images can be far more cost-effective than the use of multispectral or hyperspectral cameras. Also, RGB-based data acquisition and analyses are transferable to smart phones that are usually affordable to farmers.

The underpinnings of any successful detection model are the quality and quantity of training data. Image data in particular can vary greatly in field environments due to occlusions of the disease regions due to neighboring leaves or stems; out-of-focus target regions due to movement of the sensor or the target leaves themselves; illumination variation due to the season, time of the day, and angle of imaging; and morphological variations of leaves in terms of size, shape, and texture. Therefore, a significant contribution of this work is in the collection of diverse field images of potato across different geographies and time instants to ensure a heterogeneous training data.

Modern cell phone cameras have improved in their imaging capability along with the software-enhanced image processing offered by such phones. The acquisition of data using a variety of cell phones might lead to a model that can find wide applicability when many farmers are hesitant to adopt/invest in aerial or ground-based phenotyping equipment. Apart from model performance, inference time and memory space utilization are also important metrics for smart phone application. Therefore, the challenge will be to reduce the model size, while retaining its performance. Inference from a single image presently takes about 1-2 seconds, which makes it of practical value.

In practice, the farmer will need alerts of even a single occurrence of blight to contain it in the initial stage. In this context, it may be noted that the mAP and mAR scores provided in Table 3 are quite conservative due to the underlying concept of IoU. While evaluating the performance of the model, a detection is considered correct only if the model is able to place a bounding box around the disease that has at least 50% area of intersection with a ground-truth box demarcated by an expert. While this provides good standardization for model evaluation across various application domains, it may be noted that in the context of disease detection, the performance of the model is gauged depending on how the expert annotates the ground truth.  Figure 8, on the other hand, gives a more liberal interpretation of the model performance by testing how well the model can demarcate the disease without reference to the specific ground truth annotations. Thus, the results presented in Figure 8 show that the model presented in this work can lead to more optimistic outcomes for the potato farmer.

## 5. Conclusions

This work has demonstrated a potato blight detection model using the deep learning approach that can be applied in field conditions, for aiding the farmer in making real-time decisions. In order to improve the detection performance of the model on data acquired from easily available RGB sensors, the input data are mathematically transformed to other color spaces to aid the training of the Mask R-CNN model. It is observed that training in the LAB color space provides the highest performance metrics with 80.1% and 81.4% mAP for the 2-class and 4-class detection models, respectively. The XYZ color space has the lowest mAP values for both detection models, yielding 77.4% and 70.8%, respectively. However, the model can provide an optimistic performance of ~98% overall precision for disease detection in the real-world scenario. The feature maps of intermediate layers of the trained detection models were observed, and it was found that color spaces with better performance enabled the model to learn fine features of the disease patch, the leaf patch, and the soil patch such as color, texture, leaf venation, and leaf shape. The inference time per image and size of the detection models allow quick response when deployed in the field. This work could be extended to gauge the disease severity by quantifying the number and the size of blight disease patches per leaf.

## Figures and Tables

**Table 1:** Description of manually annotated patches for different features in the dataset.

Data	Train	Test	Total
No. of images	1423	417	1840
No. of blight patches	4673	1152	5825
No. of infected leaves	1423	356	1779
No. of healthy leaves	122	89	211

**Table 2:** Performance of various color spaces compared to RGB for potato disease detection.

Color space	2-class detection model		4-class detection model		Avg. inference time/image (sec)
	mAP (%)	mAR (%)	mAP (%)	mAR (%)	
RGB	77.4	53.9	80.9	55.5	1.77
XYZ	77.4	54.2	70.8	52.9	1.68
HSL	78.2	55.2	81.3	56.9	1.67
HSV	75.5	53.9	75.5	55.4	1.67
LAB	80.1	55.6	81.4	56.4	1.68
YCrCb	76.1	54.3	79.1	56.4	1.70

Performance score calculated for IoU = 0.5.

**Table 3:** Manually obtained performance metrics of the four-class Mask R-CNN model.

Color space	Disease patch				Infected leaf			Healthy leaf			Combined	
	TP1	TP2	FN	FP	TP	FN	FP	TP	FN	FP	*P* (%)	*R* (%)
RGB	375	166	176	7	329	22	10	93	15	2	98.1	81.9
XYZ	343	191	173	1	340	11	34	52	55	1	96.3	79.5
HSL	464	159	147	5	338	13	21	83	25	2	97.5	85.0
HSV	428	149	140	7	341	10	23	68	40	1	96.9	83.8
LAB	395	175	180	2	333	18	11	91	17	1	98.6	82.2
YCrCb	394	253	101	9	336	15	30	52	56	0	96.4	85.8

## Data Availability

All data used to train and test the model presented in this paper is freely available upon request.
